# Cytomegalovirus Infections in the Atypical Host: A Case Series of Clinical Conundra

**DOI:** 10.7759/cureus.43578

**Published:** 2023-08-16

**Authors:** Kowthar S Hassan, Ayat Idris, Batool S Al Lawati, Abdullah Balkhair

**Affiliations:** 1 Medicine, Sultan Qaboos University Hospital, Muscat, OMN; 2 Medicine/Infectious Diseases, Sultan Qaboos University Hospital, Muscat, OMN; 3 Rheumatology, Sultan Qaboos University Hospital, Muscat, OMN; 4 Infectious Diseases, Sultan Qaboos University Hospital, Muscat, OMN

**Keywords:** ganciclovir, immunodeficiencies, inflammatory bowel disease, elderly, sle, cmv infection, cmv disease

## Abstract

Background

Cytomegalovirus (CMV) reactivation may occur as the shedding of the virus from various body sites or could represent an active disease that might be fatal if untreated. Distinguishing between the two states may prove very difficult. The role of the CMV disease in patients with hematological malignancies or transplant patients is more defined than that in other immunocompromised patients where neither anti-CMV prophylaxis is used nor plasma CMV levels are monitored. Here, we try to examine cases with CMV viremia in the latter group of patients in an attempt to make a distinction between CMV infection and disease to determine which patients would benefit from treatment.

Methods

Elderly patients, patients with rheumatological disorders, and patients with inflammatory bowel disease (IBD) and with clinical suspicion of CMV disease who were referred to the infectious diseases service at Sultan Qaboos University Hospital were examined from 1 January 2018 to 31 January 2023. We added a patient we found in our referral log book from 2012. Clinical, epidemiological, and laboratory data were retrieved from the hospital information system. Plasma CMV levels and CMV body fluid levels including pulmonary samples obtained from bronchoalveolar lavage (BAL) in suspected cases of CMV pneumonitis and gastrointestinal (GI) CMV levels obtained from stool and gastrointestinal tissue biopsies in suspected cases of gastrointestinal CMV disease were collected. COBAS^®^ AmpliPrep/COBAS^®^ TaqMan^®^assay (Roche Molecular Systems, Inc., Branchburg, NJ) was used to measure CMV copies per milliliter.

Results

A total of 28 patients were considered to have CMV disease, 12 of whom were elderly (≥60 years) and the rest were young and middle aged (Y/M). The most common comorbidities of the elderly included chronic kidney disease (CKD), hypertension (HTN), and diabetes mellitus (DM). In the Y/M group, seven patients had systemic lupus erythematosus (SLE), one had antineutrophil cytoplasmic antibody (ANCA) associated vasculitis, four patients had IBD, two had IBD plus primary immunodeficiencies (one patient had agammaglobulinemia and one had combined deficiencies), and one patient had combined immunodeficiency. CKD was a common finding in the SLE patients. Diarrhea was the most common CMV presentation occurring in 19 patients (67.9%), being bloody in 10 patients. Four patients had pulmonary presentations, and four had hematological presentations in the form of anemia or pancytopenia. Nineteen patients were given CMV antiviral treatment, and one patient received it during the first episode but not in the second episode. Twenty-eight-day mortality in the treated group was 20% versus 55.5% in the untreated group. The majority of the deaths occurred in the SLE and elderly patients. Thrombocytopenia occurred in 60.7%, 70.6% of whom died signaling a potential predictive role for thrombocytopenia in early empirical CMV antiviral treatment and in prognosis.

Conclusion

The difficulty in distinguishing CMV infection from CMV disease remains a concern in the elderly and SLE patients. In our small study, there was a survival benefit in early screening for CMV and initiating preemptive CMV antiviral therapy in these two groups even before CMV disease is proven. This urgency was not observed for patients with IBD or primary immunodeficiencies. A major common factor for CMV disease was CKD, whereas thrombocytopenia was an indicator of disease and prognosis.

## Introduction

Cytomegalovirus (CMV) is one of the human betaherpesviruses, which usually infect humans in childhood leading to high seropositivity in the adult population globally [1,2]. The primary infection is usually subclinical or causes a mild infectious mononucleosis-like illness [3]. Like other herpesviruses, CMV establishes latency post primary infection, and the latent virus may reactivate if the immune system is compromised with resultant viral shedding [4]. The detected viral levels could represent the shedding of the virus, which requires no intervention or may represent CMV disease that requires active CMV antiviral treatment without which it could be fatal. There are a number of factors that make the distinction between CMV infection and CMV diseases difficult especially in patients who are not traditionally known as "at-risk patients." A major factor is the lack of correlation between serum levels of CMV and end-organ levels as negative serum CMV does not rule out end-organ disease. Furthermore, the mere presence of CMV in body fluids of any organ does not satisfy a diagnosis of CMV disease whereby the demonstration of the typical cytopathic effects of CMV in the form of inclusion bodies (owl eyes) is required [5]. Moreover, the sampling of body fluids such as bronchoalveolar lavage (BAL) and gastrointestinal (GI) endoscopy for CMV quantification and tissue sampling add to the burden of making a timely diagnosis in critically ill patients. Lastly, presentations such as diarrhea and pneumonia might be common to the disease background making the differentiation from CMV disease very challenging.

## Materials and methods

Elderly patients (>60 years according to the United Nations {UN}) [6], patients with rheumatological disorders, and patients with inflammatory bowel disease (IBD) and with clinical suspicion of CMV disease who were referred to the infectious diseases service at Sultan Qaboos University Hospital were examined from 1 January 2018 to 31 January 2023. We added a patient we found in our referral log book from 2012. Clinical, epidemiological, and laboratory data were retrieved from the hospital's electronic medical records. Plasma CMV levels and CMV body fluid levels including pulmonary samples obtained from BAL in suspected cases of CMV pneumonitis and gastrointestinal CMV levels obtained from stool and gastrointestinal tissue biopsies in suspected cases of gastrointestinal CMV disease were collected. Quantitative CMV polymerase chain reaction (PCR) determinations of greater than 1000 copies per milliliter was considered significant [7]. COBAS® AmpliPrep/COBAS® TaqMan® assay (Roche Molecular Systems, Inc., Branchburg, NJ) was used to measure CMV copies per milliliter.

Ethical approval was obtained from the Sultan Qaboos University Hospital Medical Research Ethics Committee (MREC #2917).

## Results

We identified a total of 28 patients in whom we considered CMV disease as a potential cause for their condition (20 females and eight males) with a median age of 47 years (minimum, 26 years; maximum, 90 years). There were 12 elderly patients (age of >60 years) with various multiple comorbidities including hypertension (HTN), diabetes mellitus (DM), chronic kidney disease/end-stage renal disease (CKD/ESRD), heart failure (HF), liver cirrhosis, and rheumatoid arthritis (RA) and one patient with renal transplant and antineutrophil cytoplasmic antibody (ANCA)-associated vasculitis. In the young and middle age (Y/M) groups, there were 16 patients, seven of which had systemic lupus erythematosus (SLE), one had ANCA-associated vasculitis, five had IBD, three had primary immunodeficiencies (one of whom also had IBD), and one had DM with ESRD (Table 1).

**Table 1:** Clinical characteristics of 28 patients suspected to have CMV disease SLE, systemic lupus erythematosus; CKD, chronic kidney disease; SCT, sickle cell trait; ESRD, end-stage renal disease; HD, hemodialysis; APL, antiphospholipid syndrome; HTN, hypertension; PHTN, pulmonary hypertension; RA, rheumatoid arthritis; IHD, ischemic heart disease; BPH, benign prostatic hypertrophy; DM, diabetes mellitus; BSI, bloodstream infection; MDRA, multidrug-resistant *Acinetobacter*; VRE, vancomycin-resistant *Enterococcus*; BAL, bronchoalveolar lavage; CMV, cytomegalovirus; CRP, C-reactive protein; GI, gastrointestinal; HF, heart failure; ANCA, antineutrophil cytoplasmic antibody; AKI, acute kidney injury

Age/sex	Primary diagnoses	CMV syndrome presentation	Pretreatment peak plasma CMV (copies/mL)	Stool CMV (copies/mL)	Tissue/body fluid CMV (copies/mL)	Histopathology	Peak CRP (mg/L)	Nadir platelets (×10^9^/L)	CMV treatment	Outcome
26/female	SLE with nephritis	Diarrhea and cytopenia	37750	Not done	Not done	Not done	37	44	Ganciclovir	Died of VRE BSI
48/female	SLE, DM, and CKD,	Diarrhea	20953	151820	Not done	Sigmoid ulceration CMV inclusions seen. CMV stain positive	27	253	Ganciclovir	Alive
55/female	SLE, APL, CKD, and Graves' disease	Fever and abdominal pain	<150 copies/mL	Not done	>10 million (esophagus)	Esophagus: inflamed granulation tissue and fibrinous exudate. CMV inclusions seen. CMV stain positive	222	76	No anti-CMV treatment	Died of massive GI bleed
44/female	SLE, HD, HF, and PHTN	Odynophagia and dyspnea	60323	Not done	Not done	Duodenum: no CMV features. CMV stain positive	32	208	Ganciclovir	Died of bowel ischemia
34/female	SLE	Pneumonitis	399	1094	4573 (BAL)	Not done	51	25	Ganciclovir followed by foscarnet	Died of alveolar hemorrhage
40/female	SLE, APL, CKD, HF, and HTN	Alveolar hemorrhage	4014	Not done	2586216 (BAL)	Not done	45	84	Ganciclovir	Alive
31/female	SLE, CKD, and HF	Anemia and thrombocytopenia	11138	Not done	Not done	Not done	68	96	Ganciclovir	Died
45/female	ANCA vasculitis and ESRD	Pancytopenia	540638	Not done	Not done	Not done	8	55	Ganciclovir	Alive
30/female	Ulcerative colitis	Bloody diarrhea	<150	5323	264171 (rectal)	Rectum: cryptic distortion of ulcerative colitis (UC). Sigmoid: CMV inclusions seen	136	453	Ganciclovir	Alive
31/female	Ulcerative colitis	Abdominal pain and bloody diarrhea	181	477	<150 (colon)	Sigmoid: nonspecific inflammation. CMV stain negative	103	269	Ganciclovir	Alive
37/male	Ulcerative colitis	Abdominal pain and bloody diarrhea	<150	Not done	<150 (colon)	Colon: cryptic distortion with abscesses. CMV inclusions seen	17	347	Ganciclovir followed by oral valganciclovir	Alive
40/female	Ulcerative colitis	Bloody diarrhea	<150	Not done	138296 (colon)	Colon: multiple ulcers and cryptitis with abscesses. CMV inclusions seen	31	352	No CMV antiviral treatment	Alive
30/female	Ulcerative colitis, DM, and combined immunodeficiency	Diarrhea	<150	Not done	44446 (colon)	Colon: crypt distortion with abscess. Moderate active colitis with features of CMV. CMV inclusions negative. CMV stain positive	24/11/21: 282	24/11: 396	No CMV antiviral treatment	Alive
29/male	Combined immunodeficiency	Intermittent diarrhea	Not done	Not done	29895 (colon)	Colon: cryptitis. CMV inclusions negative. CMV stain negative	11	136	No CMV antiviral treatment	Alive
41/male	Ulcerative colitis and hypogammaglobulinemia	Chronic diarrhea	<150	<150	57900 (colon)	Colon: cryptitis with abscesses. CMV inclusions negative. CMV stain negative	36	213	Ganciclovir	Alive
47/female	DM, SCT, and ESRD (HD)	Diarrhea	11729	Not done	Not done	Not done	7	224	Ganciclovir	Died of pulmonary embolism
60/male	ANCA vasculitis and renal transplant	Anemia	455	Not done	Not done	Not done	381	65	No CMV antiviral treatment	Died of MDRA BSI
61/female	DM, HTN, ESRD (HD), and necrotizing fasciitis of the breast	Diarrhea	121206	<150	192 (colon)	Colon: intact architecture and no ulceration. CMV inclusions negative. CMV stain negative	28	90	Foscarnet	Died
64/female	Renal transplant (failed), ESRD (HD), and amyloid	Diarrhea	4665	Not done	Not done	Colon: acute and chronic inflammation. CMV inclusions positive	6	70	Ganciclovir	Died
69/female	HTN and RA	Fever, abdominal pain, and bloody diarrhea	<150	Not done	1991794 (colon)	Colon: focal ulceration with granulation tissue. CMV stain positive	72	144	Ganciclovir	Alive
69/female	HTN, hypothyroid, and AKI	Bloody diarrhea	11455	Not done	>10 million (colon)	Colon: ulceration with granulation tissue. CMV inclusions positive	112	129	Ganciclovir	Died of BSI
72/female	DM, HTN, and IHD	Bloody diarrhea, fever, and cholecystitis	10543	1448	Not done	Not done	64	393	Ganciclovir	Died
80/male	Alzheimer's disease and BPH	Fever, abdominal pain, and rectal bleeding	4599	<150	Not done	Colon: ulceration with granulation tissue. CMV stain positive	50	332	Ganciclovir	Died of *Candida auris* candidemia
81/male	DM, HTN, and hypothyroidism	Respiratory failure	17175	Not done	1080201 (BAL)	Not done	126	145	Foscarnet	Died of MDRA BSI
81/male	DM, HTN, CKD, and hypothyroidism	Bloody diarrhea	8174	38296	Not done	Not done	81	86	Ganciclovir followed by valganciclovir	Alive
82/male	DM, HTN CKD, and liver abscess	Diarrhea	9310	Not done	Not done	Not done	148	12/9: 109	No CMV antiviral treatment	Died
85/female	HTN, cirrhosis, and asthma	Diarrhea and upper GI bleed	1876	1870	Not done	Not done	51	11	No CMV antiviral treatment	Died
90/female	DM, HTN, CKD, and HF	Diarrhea	<150	Not done	1595 (colon)	Colon: multiple ulceration and granulation tissue. CMV stain negative	32	60	No CMV antiviral treatment	Died of severe *Clostridioides difficile* infection

High levels of CMV (>1000 copies/mL) were detected in the plasma of 15 patients (range: 1617-540638 copies/mL); lower levels (<1000 copies/mL) were seen in three patients (183, 399, and 455 copies/mL, respectively). The remaining 10 patients had plasma CMV viral load of <150 copies/mL. Among patients with diarrhea, 11 patients had high CMV levels in their plasma, five had low levels, CMV in plasma was not done in one patient, and the rest had negative CMV in plasma. Ten patients had high CMV levels in their gastrointestinal tissues (esophagus to sigmoid). One patient did not have CMV measured in stools and tissues nor had biopsy done, but her plasma CMV reached 37750 copies/mL. Inclusion bodies were positive in five patients, two of whom were also positive for immunohistochemical staining and one negative and staining was not done in two. Six patients were negative for inclusion bodies with one patient having positive staining, three having negative staining, and staining not mentioned in two. For the rest of the patients, biopsy report did not mention inclusions or staining findings.

Among the four patients with dyspnea and suspected CMV pneumonitis, two had low CMV levels in plasma but high in BAL, and two had high levels in both plasma and BAL. Twenty patients (71.4%) received treatment (ganciclovir or foscarnet). The main CMV presentation was diarrhea (n=19, 68%) that was bloody in 10 patients and associated with abdominal cramps in some. Other presentations included dyspnea that occurred in four patients (14.3%) and anemia or pancytopenia. Deranged liver function tests (LFTs) were seen in four patients. CRP ranged from 8 to 328 mg/L, and platelets were low in 17 patients (60.7%), 12 (70.6%) of whom died. A total of 16 patients expired (57%), six patients from the untreated group and 10 from the treated group. In the untreated group, one died from massive gastrointestinal bleed, one from a multidrug-resistant organism (MDRO) bloodstream infection (BSI), one from recurrent severe *Clostridioides difficile* infection, and two with septic shock but negative blood cultures. The sixth patient also died of sepsis with negative blood cultures but had been treated for CMV in the first episode, which he survived but not the second time. In the treated group, one patient died from diffuse alveolar hemorrhage due to SLE disease activity, one from bowel ischemia due to active SLE, one from massive pulmonary embolism, three from MDRO BSI, one from *Candida auris* BSI, one from respiratory acidosis due to accidental blockage of endotracheal tube, one from citrate toxicity, and one from sepsis with negative blood cultures. Median time to death from receiving anti-CMV treatment was 36 days (minimum, two days; maximum, 468 days) and 43 days from the first high CMV level (minimum, three days; maximum, 110 days). In the untreated group, median time to death was three days (minimum, zero; maximum, 125 days) from the first high CMV level; one patient died before CMV result level was obtained. Only three patients in the untreated group survived. Mortality at day 28 from starting ganciclovir was 20% from the first high CMV in the treated group. In the untreated group, 28-day mortality was 55.5% from first high CMV.

## Discussion

CMV is one of the herpesviruses that has a wide spectrum of clinical manifestations ranging from subclinical picture to fatal outcome depending on the immune status of the host [8]. CMV disease is further divided into CMV syndrome characterized by fever, myalgias, arthralgias, and cytopenia or tissue invasion where inclusion bodies are found in involved tissues [9]. Such patients are usually started on CMV prophylaxis or undergo weekly measurements of serum CMV levels as a marker for the disease, which alarms for the initiation of therapy. Similar protocols, however, are not followed for other immunocompromised patients such as patients with SLE or the elderly. Our interest in reviewing this subject was triggered by the death of a young female with well-controlled SLE, from a sudden massive gastrointestinal bleed. She had presented with two days of fever, abdominal pain, and four days of constipation and had negative serum CMV (she was not viremic when tested). She underwent upper gastrointestinal endoscopy, the tissue of which showed millions of CMV copies and inclusion bodies. Despite her controlled SLE, she had unexplained thrombocytopenia for 13 months before her last presentation. Moreover, we had observed, over a number of previous years, some elderly bed-bound patients presenting with bloody diarrhea with abdominal CT showing colitis but an unestablished diagnosis ending in fatal outcomes. We embarked on this study to better understand the difference between CMV infection and CMV disease in this group of patients and better inform future decisions.

In the elderly group, a number of studies have investigated the association between CMV, immunity, and mortality in old age. CMV infection was shown to be associated with expanded CMV-specific cluster of differentiation 8 (CD8) cells and diminished immunity to other infections due to a decrease in immune cell function and differentiation [10]. High CMV antibody titer in the elderly has been linked to increased mortality, and the excess of vascular death in healthy elderly was also blamed on CMV infection [11,12]. However, such studies did not address the question of CMV disease in the elderly and its distinction from CMV infection. In our study, there were 12 elderly patients with various comorbidities presenting mainly with diarrhea occurring in 10 patients, which was bloody in five, and one with upper gastrointestinal bleed. These patients were investigated for *Clostridioides difficile* infection, and CMV was requested in blood and stools as part of the initial investigations. Although six of the treated patients expired, the cause of death was not CMV-related. One died on day 2 of anti-CMV initiation, which could be due to a delay in treatment. One of the untreated patients had severe *C. difficile* infection requiring treatment with oral vancomycin and metronidazole. She eventually died from *C. difficile* infection on day 125 post initial presentation. As CMV infection in the elderly is known to reduce immunity, it might have contributed to the recurrent eventually fatal *C. difficile* infection. ESRD/CKD was also prevalent in this group, being present in five patients, and in two patients, acute kidney injury (AKI) developed.

In the Y/M group (n=16), eight patients had rheumatological conditions, mainly SLE in seven and ANCA-associated vasculitis in one, all of whom had ESRD or CKD. One patient with DM and sickle cell trait (SCT) also had ESRD. This is in agreement with the risk factors for CMV disease [13]. The spectrum of gastrointestinal presentation is in agreement with a review by Rafailidis [14]. Diarrhea in patients with SLE could be attributed to disease flare, medications such as mycophenolate mofetil, or gastrointestinal opportunistic infection. In three of the four SLE patients that presented with gastrointestinal symptoms, biopsy proved CMV disease by demonstrating inclusion bodies and by immunohistochemistry (IHC) staining. This is in agreement with Guta et al. who demonstrated active CMV or Epstein-Barr virus disease in one-third of their patients with SLE [15]. Undetected plasma CMV levels can be deceptive. This is consistent with the fact about dissociation between the plasma and the end-organ CMV levels. Therefore, we agree with the suggestion of Sebastiani et al. for early screening for CMV not only in plasma but also in stools and gastrointestinal tissue, histology by both inclusion bodies, and immunohistochemical staining [16]. We also advocate for early empirical treatment for CMV in high-risk patients if not contraindicated while CMV biopsy results are awaited. In patient 4, the first biopsy reported no features of CMV disease without specifying the methods of staining used. However, inclusion bodies were later seen in the second biopsy. CMV inclusion bodies are demonstrated with hematoxylin and eosin staining, which has a lower sensitivity than the immunohistochemical staining, which is gold standard [17]. Currently, there are no clear guidelines as to the administration of the immunosuppressive agents during active CMV infection in immunocompromised patients. Diagnosing CMV disease of the lung might be even more difficult than CMV gastrointestinal disease due to difficulty in obtaining tissue (lung) biopsy. Two patients with CMV pneumonitis were very noncompliant with SLE treatment. They both died despite receiving CMV antiviral treatment while their immunosuppressive agents had been discontinued. Three patient received anti-CMV treatment and survived. This again warrants continuing immunosuppressive agents while on treatment for CMV.

The difficulty in diagnosing CMV colitis in IBD is also very challenging as bloody diarrhea could be due to the inflammatory bowel disease itself. do Carmo et al. had shown that latent CMV was common in patients with IBD, active CMV disease was rare, and there was no association between active CMV infection and IBD activity [18]. On the other hand, CMV colitis linked to unfavorable outcome was found to be more associated with IBD causing clinical and endoscopic findings undistinguishable from those of IBD and relying solely on demonstration on cytopathic effects of the virus [19]. In our study, of the five patients with IBD, three had features of CMV colitis in the form of inclusion bodies or positive immunohistochemical staining, and two were negative despite having high tissue CMV PCR levels. To date, all the patients with IBD and CMV are alive (treated or untreated for CMV). The ones that did not receive CMV antiviral treatment responded to their IBD treatment but had other relapses of the diarrhea. One of the patients had hypogammaglobulinemia and IBD, with previous giardia and salmonella infections. He was not responding to immunosuppressive agents nor to anti-giardia combination treatment. Upon reviewing his pathology, occasional inclusion bodies were reported in a tissue biopsy five months earlier with negative immunohistochemical stain and a negative biopsy in the current visit. Upon the commencement of ganciclovir, his diarrhea completely resolved. This case is difficult to explain from the prospective of responding to ganciclovir despite the current biopsy being negative for inclusion bodies and IHC stain unless if the biopsy missed the affected parts. Although the number of patients with IBD and CMV in our cohort is very small, it does, however, seem that waiting for a biopsy proof of CMV disease is feasible unlike the cases of SLE.

We also looked at other parameters including C-reactive protein (CRP), platelets, and LFT in an attempt to distinguish between CMV infection and CMV disease to decide upon empirical anti-CMV treatment. LFTs were not helpful as mild derangement was seen only in four patients. CRP levels were also not helpful in distinguishing between the two states of the virus ranging from 6 to 381 mg/L. On the other hand, 17 patients (60.7%) had low platelets, 12 (70.6%) of whom died indicating a potential use of thrombocytopenia as an indicator of CMV disease and a prognostic factor for mortality. Patient 3 had thrombocytopenia fluctuating between 29 and 140 for 13 months before her final presentation that was fatal. Thrombocytopenia has been reported in association with CMV infection and was noted to require CMV antiviral treatment as a response to steroids is not adequate [20,21].

We noted a hesitancy in starting CMV antiviral treatment while awaiting CMV tissue sampling. We, however, discourage this hesitancy when this group of at-risk patients presents with manifestations compatible with CMV disease and has high levels of CMV in plasma or body fluids. Overall, in the SLE patients and the elderly group, there is a clear benefit of treatment as evidenced by the 28-day mortality being 20% versus 55.5% in the treated and untreated groups, respectively.

We faced a number of challenges in this study including the lack of standard protocols regarding when and in which patients to suspect CMV disease, ordering the appropriate samples, and standardized histopathology reporting. We took the initiative to set a protocol to solve these issues. We are planning to recruit more patients for a bigger study by setting up a protocol that involves the elderly and patients with SLE.

Limitations

The main limitation of this case series is the small number of patients and the mixture of patients grouped in one case series. Another major limitation is the lack of a structured protocol regarding the inclusion of the patients. Patients were mainly from our personal list that we had encountered but probably were not referred to us. Furthermore, the study lacked standards regarding sampling and pathology reporting.

## Conclusions

This study suggests that in the elderly and patients with SLE, especially those with CKD or ESRD, early screening for CMV infection should be carried out, and empirical treatment with CMV antiviral therapy while waiting for confirmation should be considered when patients present with CMV-compatible syndromes. On the other hand, more time is afforded to rule out CMV disease in patients with IBD. Thrombocytopenia could be useful as an indicator of CMV disease and its prognosis.

As distinguishing between CMV infection and disease could be at times impossible, perhaps a new strategy should be explored such as the use of proteomics on the assumption that different proteins are secreted by the reactivated latent and active lytic virus.
